# An androgen response element driven reporter assay for the detection of androgen receptor activity in prostate cells

**DOI:** 10.1371/journal.pone.0177861

**Published:** 2017-06-01

**Authors:** Waqas Azeem, Margrete Reime Hellem, Jan Roger Olsen, Yaping Hua, Kristo Marvyin, Yi Qu, Biaoyang Lin, Xisong Ke, Anne Margrete Øyan, Karl-Henning Kalland

**Affiliations:** 1 Department of Clinical Science, University of Bergen, Bergen, Norway; 2 Centre for Cancer Biomarkers, University of Bergen, Bergen, Norway; 3 Department of Microbiology, Haukeland University Hospital, Bergen, Norway; 4 Zhejiang California International NanoSystems Institute, Hangzhou, P.R. China; 5 Department of Urology, University of Washington, Seattle, Washington, United States of America; Northern Institute for Cancer Research, UNITED KINGDOM

## Abstract

The androgen receptor (AR) transcription factor plays a key role in the development and progression of prostate cancer, as is evident from the efficacy of androgen-deprivation therapy, AR is also the most frequently mutated gene, in castration resistant prostate cancer (CRPC). AR has therefore become an even more attractive therapeutic target in aggressive and disseminated prostate cancer. To investigate mechanisms of AR and AR target gene activation in different subpopulations of prostate cancer cells, a toolkit of AR expressor and androgen response element (ARE) reporter vectors were developed. Three ARE reporter vectors were constructed with different ARE consensus sequences in promoters linked to either fluorescence or luciferase reporter genes in lentiviral vector backbones. Cell lines transduced with the different vectors expressed the reporters in an androgen-dependent way according to fluorescence microscopy, flow cytometry and multi-well fluorescent and luminescence assays. Interestingly, the background reporter activity in androgen-depleted medium was significantly higher in LNCaP cells compared to the prostate transit amplifying epithelial cell lines, EP156T-AR and 957E/hTERT-AR with exogenous AR. The androgen-induced signal to background was much higher in the latter benign prostate cells than in LNCaP cells. Androgen-independent nuclear localization of AR was seen in LNCaP cells and reduced ARE-signaling was seen following treatment with abiraterone, an androgen synthesis inhibitor. The ARE reporter activity was significantly stronger when stimulated by androgens than by β-estradiol, progesterone and dexamethasone in all tested cell types. Finally, no androgen-induced ARE reporter activity was observed in tumorigenic mesenchymal progeny cells of EP156T cells following epithelial to mesenchymal transition. This underscores the observation that expression of the classical luminal differentiation transcriptome is restricted in mesenchymal type cells with or without AR expression, and presence of androgen.

## Introduction

Androgen receptor (AR) plays a critical role in the normal development and function of the prostate gland [1]. AR, also named NR3C4 (nuclear receptor subfamily 3, group C, member 4), belongs to the steroid hormone group of nuclear receptors [2]. It has four distinct structural and functional domains, a less conserved N-terminal domain (NTD), the central highly conserved DNA-binding domain (DBD) which is connected to the moderately conserved C-terminal ligand binding domain (LBD) by a flexible hinge region [3]. AR is a ligand-dependent transcription factor that is present in the cytoplasm in close association with heat shock proteins (HSPs) when inactive [4]. Binding to its native ligands, testosterone or 5α-dihydrotestosterone (DHT), induces nuclear translocation of AR by a series of conformational changes that lead to displacement of HSPs and binding of AR to importin-α [5]. The activity of AR is regulated by interactions between the AR functional domains and cofactors. The interactions between AR functional domains, the NTD-LBD intra- and interdomain interactions, and the DBD-DBD interdomain interaction, facilitate AR dimerization [6]. In the nucleus, the AR homodimer recruits a variety of co-regulatory proteins and binds to specific DNA sequences, termed androgen response elements (ARE), in the promoter and enhancer regions of target genes, such as prostate-specific antigen (PSA) and transmembrane protease serine 2 (TMPRSS2), and thus modulates the transcription of androgen responsive genes [7–10]. The ARE consists of the consensus sequence for AR binding in two equal, hexameric half-sites organized in direct repeats formation, separated by a three base-pairs spacer (IR3) [11].

Prostate cancer is the most frequently diagnosed cancer and the second most common cause of cancer death in men [12]. AR plays an important role in the development and progression of prostate cancer. AR remains functional and expressed in nearly all primary prostate cancers [13]. At initial stages, almost all prostate cancers are dependent on androgenic stimulation. Locally advanced or metastatic prostate cancers are initially treated by androgen deprivation therapy (ADT), thereby reducing AR activation. However, the effect of ADT is transient, and patients ultimately develop castration-resistant prostate cancer (CRPC) [14, 15]. AR activity can be maintained in CRPC by mutation or amplification of AR or local production of androgens. It has also been shown that AR can promote CRPC development through the activation of AR ligand-independent pathways discrete from AR-ligand dependent pathways [16–20]. As AR activity remains important in the progression of all stages of prostate cancer, AR continues to be an attractive molecular target of drugs against prostate cancer. A major problem in treatment targeting the AR-signaling axis is the eventual development of drug resistance [21, 22], therefore new drugs targeting AR-signaling would be beneficial.

In our study, we developed ARE based assays that are useful to understand the AR activity in different conditions. The reporter assays are helpful in the evaluation of potential drug candidates in AR-directed therapies. The assays provide much faster ways to test the AR activity on a large scale. Additionally, they can be useful to study therapeutic efficacy and development of drug resistance *in vivo* and in *in vitro* three-dimensional (3D) tumor models.

## Materials and methods

### Cell lines and cell cultures

The prostate transit amplifying basal epithelial cell line EP156T was grown in MCDB153 medium (Biological Ind. Ltd., Israel) with 1% fetal calf serum (FCS) and supplemented with growth factors and antibiotic [23]. The prostate cancer cell line LNCaP (ATCC-CRL-1740, LGC Standards GmbH, Wesel, Germany) was grown in RPMI-1640 (Lonza, Verviers, Belgium) with 10% FCS. 957E/hTERT-AR cells were provided by John T. Isaacs lab (Johns Hopkins, MD, USA) were grown in Keratinocyte SFM (Thermo Fischer Scientific, MA, USA) [24]. EPT3-PT1 and EPT3-PT1-AR cells were grown in Ham’s F12 medium (Lonza, Basel, Switzerland) with 5% FCS. For experiments investigating the androgen stimulated AR response, cells were grown in androgen free medium containing charcoal-stripped FCS (CS FCS).

### Vectors

AR expression clones were generated by performing LR recombination reactions to insert the *AR* gene into destination vectors according to Invitrogen user manual (MAN0000682). The AR expression clone AR GC-E2325 (GeneCopoeia^™^, Rockville, MD, United States, GenBank Accession: NM_000044) was transferred to the destination vectors pLenti6.3/V5-DEST and pLenti7.3/V5-DEST (Invitrogen, Life Technologies, Carlsbad, CA, United States) vectors, and the AR expression clone with V5 tag AR GC-E0060-CF (GeneCopoeia^™^, Rockville, MD, United States, Accession: AF162704) was transferred to pLenti6.3/V5-DEST destination vector. The schematic maps of AR expression vectors are shown in S2 Fig.

ARE reporter vectors were developed by inserting ARE consensus binding artificial promoter combinations in the 3^rd^ generation lentiviral destination vector pEZ-Lv152 (GeneCopoeia^™^, Rockville, MD, United States). Three ARE reporter vectors CS-GS241B-gLUC-Lv152, CS-GS248B-gLUC-Lv152 and CS-GS249B-gLUC-Lv152 with Gaussia luciferase reporter signals and three ARE reporter vectors CS-GS241B-mCHER-LV152, CS-GS241B-mCHER-LV207-01 and CS-GS241B-mCHER-LV207-02 with mCherry fluorescent reporter signals were prepared and supplied by Labomics S.A. (Nivelles, Belgium). The schematics of ARE reporter vectors are shown in S1 Fig.

### Transfections and Transductions

#### Generation of exogenous AR expression in prostate basal epithelial cells

The virus particles were generated by transfecting 293FT producer cells with pLenti6.3/AR-GC-E2325 AR expression vector and ViraPower^™^ Packaging Mix according to the manufacturer’s guideline (Invitrogen, Cat. No. K370-20). Lentivirus was harvested from the supernatant 48 hours and 72 hours post transfection. EP156T cells were infected with harvested lentiviral particles combined with polybrene at final concentration 8 μg/ml and incubated for 24 hours. Used medium was replaced with androgen-free complete MCDB153 medium containing growth factors, 1% charcoal stripped FCS for 48 hours. The infected target cells were cultivated with 2 μg/ml blasticidin, and stably transduced EP156T-AR cells were selected. EP156T-AR cells were grown and maintained in androgen-free MCDB153 medium containing growth factors and charcoal stripped 1% FCS.

#### Generation of prostate ARE reporter cell lines

The virus particles were generated by transfecting CS-GS241B-mCHER-LV152 and HIV packaging mix in HEK 293Ta lentiviral packaging cell line following the manufacturer’s instructions (GeneCopoeia^™^, Cat. No. HPK-LvTR-20). The lentiviral particles in the supernatant were harvested 48 and 72 hours post transfection and treated with Lenti-Pac^™^ Lentivirus Concentration Solution according to the manufacturer’s guideline (GeneCopoeia^™^, Cat. No. LPR-LCS-01). EP156T, EP156T-AR, 957E/hTERT-AR, EPT3-PT1-AR and LNCaP cells were infected with harvested lentiviral particles combined with polybrene at a final concentration of 8 μg/ml. Used medium was replaced with fresh medium and cells were incubated at 37°C for 48 hours. The infected target cells were grown with 12.5 μg/ml hygromycin and stably transduced EP156T-241B, EP156T-AR-241B, 957E/hTERT-AR-241B, EPT3-PT1-AR-241B and LNCaP-241B cells were selected. In addition, LNCaP cells were transduced with CS-GS241B-mCHER-LV207-01 and CS-GS241B-mCHER-LV207-02 vectors following the same protocol to obtain LNCaP-207-01 and LNCaP-207-02 cells, respectively.

### Preparation of luciferase assays

#### Preparation of exogenous AR luciferase assay

The HEK 293FT producer cells were grown in white walled, flat surface 96-well plates and co-transfected with Cignal Androgen Receptor Reporter (CCS-1019L) and AR expression vectors according to the manufacturer’s guideline (Qiagen, Venlo, Netherlands, Cat. No. CCS-1019L). Lipofectamine^™^ 2000 (Invitrogen, Cat. No. 11668–019) transfection reagent was used. The cells were maintained in androgen-free DMEM medium (Lonza, Verviers, Belgium) with 10% charcoal-stripped FCS (Sigma Aldrich, St. Louis, MO, USA), treated and were assayed for Firefly and Renilla luciferase activities in Synergy H1 Hybrid Reader (BioTek, Winooski, Vermont) using appropriate luciferase substrate from Dual-Glo^®^ Luciferase Assay System (Promega, Cat. No. E2940). All luciferase assays were performed at least in triplicate.

#### Preparation of ARE reporter response luciferase assay

The 293FT producer cells were grown in white walled, flat surface 96-well plates and were co-transfected with AR expression vector and ARE reporter vectors according to the manufacturer’s instruction (Invitrogen, Cat. No. 11668–019). The cells were maintained in androgen-free DMEM medium with 10% charcoal-stripped FCS, treated and the supernatant was monitored for Gaussia luciferase activity in a Synergy H1 Hybrid Reader (BioTek, Winooski, Vermont) using appropriate luciferase substrate from Secrete-Pair^™^ *Gaussia* Luciferase Assay Kit (GeneCopoeia. Cat. No. SPGA-G100). Afterwards, cell proliferation assays were performed using CellTiter 96^®^ AQ_ueous_ One Solution Cell Proliferation Assay (Promega, Cat. No. G3581). All the experiments were performed at least in triplicate.

### Flow cytometry

One day before the treatment, ARE responsive reporter cells were grown in androgen-free medium in 6-well plates. The cells were treated and harvested after 24 hours, washed twice with cold (4°C) PBS and immediately analysed on the LSRFortessa cytometer (BD, Biosciences, Heidelberg, Germany). All subsequent analyses were performed with FlowJo software (Tree Star, Ashland, OR, USA). One percent events were accepted as false-positive in the negative controls throughout the experiments. All the experiments were performed at least in triplicate.

### Multi-plate fluorescence reader and fluorescence microscopy

One day before the treatment, ARE responsive reporter cells were grown in androgen-free medium in black walled, flat surface 96-well plates. The cells were treated and analyzed for fluorescence signals by the Leica DM IRBM microscope (Leica Microsystems Inc, Deerfield, IL) using Qcapture software (QImaging) and Synergy H1 Hybrid Reader (BioTek, Winooski, Vermont) using Gen5 software (BioTek). The excitation was set at 584 nm and emission at 610 nm for mCherry signals.

### Indirect immunofluorescence assay

The cells were grown on 12 mm glass coverslips (Assistant, Sondheim v. d. Rhon Germany) in 24 well plates. The cells were washed with PBS and fixed with 4% formaldehyde solution in PBS for 20 min at room temperature (RT). After fixation, cells were washed twice with PBS and subjected to membrane permeabilization with 0.5% Triton X-100 for 5 min. After permeabilization, cells were washed twice with PBS and treated with 100 mM glycine in PBS for 10 min to quench free aldehyde groups and reduce background fluorescence. The cells were then washed with PBS and blocked with 0.5% BSA in PBS for 15 min at RT. Primary Anti-AR antibody (Abcam, Cat. No. 133273) was added with 1:100 dilution in 0.5% BSA in PBS and incubated for an hour at RT. Following 3 times washing with PBS, the cells were incubated with Texas red-labelled secondary anti-rabbit IgG (Southern Biotech, AL, USA) for 30 min at RT in dark. The cells were than washed twice in PBS and the coverslips were mounted in Prolong Gold with DAPI (Molecular Probes, Life Technologies, Cat. No. 535142) on glass slides and were analyzed.

### Statistical analysis

Mean values of three independent experiments with standard error of the mean (SEM) or standard deviation (SD) are displayed. Significance was confirmed by using unpaired two-tailed Student’s *t*-test. *p ≤ 0.05, **p ≤ *0*.*01*, ***p ≤ *0*.*001*, **** *p* ≤ 0.0001. Z´-factor values were calculated as described by Zhang et. al. [25] using the following equation: Z´-factor = 1-(3σ_p_+σ_n_)/|μ_p_-μ_n_| where σ_p_ and σ_n_ are the standard deviations of positive and negative controls, respectively, and μ_p_ and μ_n_ are the means of positive and negative controls, respectively. Fluorescence intensity data from the three independent experiments were used to calculate Z´-factor where 1 nM R1881 induced samples were used as positive controls and untreated ethanol (ETOH) samples as negative controls.

## Results and discussion

### AR gene transfection and expression analysis

AR remains critically important in prostate cancer as shown by the fact that although CRPC may be independent of androgen levels, it continues to rely on AR signaling. The factors that promote AR signaling in CRPC involve AR upregulation, mutations, co-activator protein alterations and tumor microenvironment effects [26–30]. Thus, not only the expression level of AR protein but also the functional activity of AR is important at all stages of prostate cancers.

**Fig 1:**
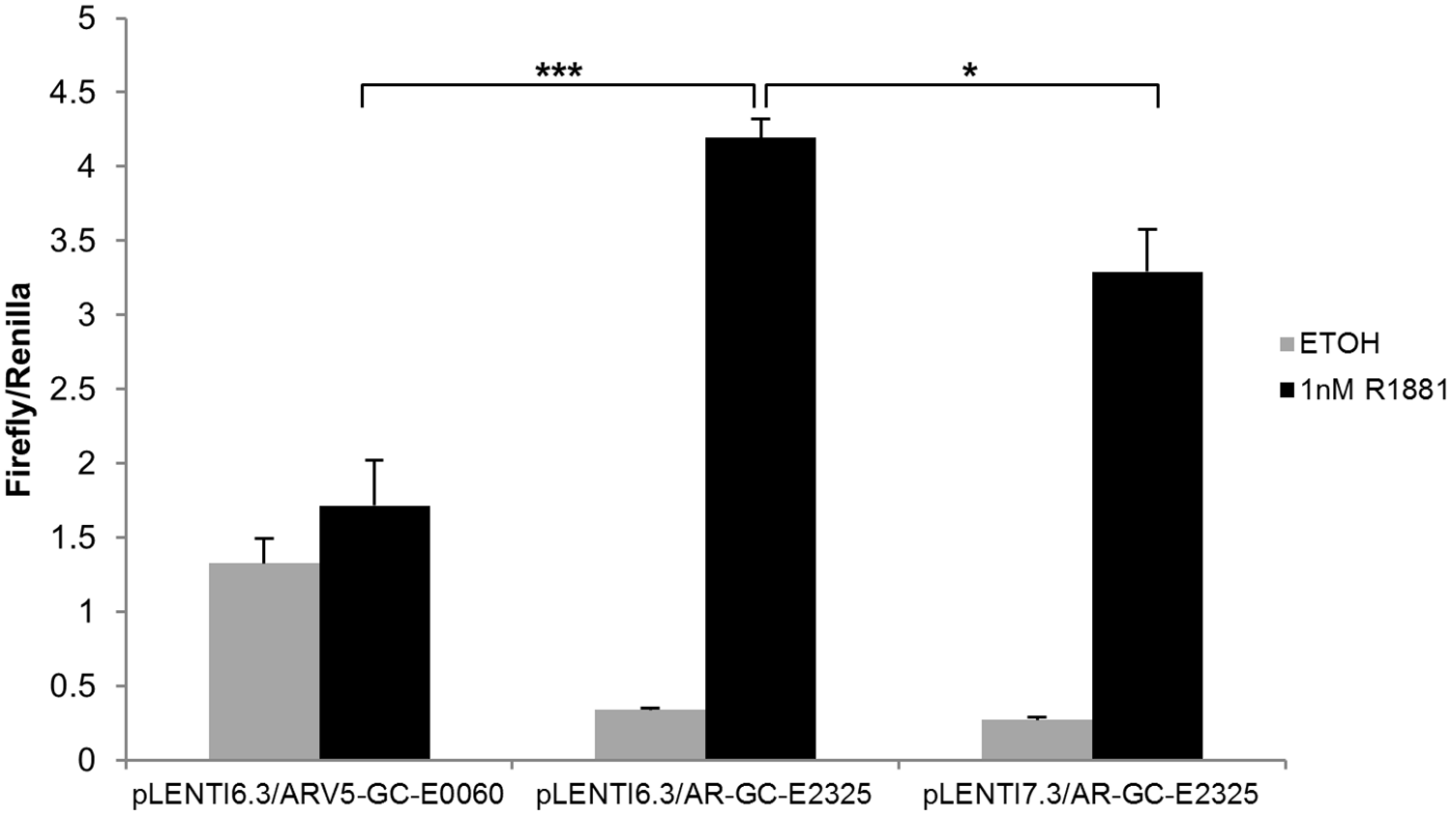
Comparison of different AR expression vectors in 293FT cells. 293FT cells were co-transfected with ARE reporter CCS-1019L and three separate AR expression vectors. The cells were grown with or without synthetic androgen 1 nM R1881 for 24 hours. The AR expression was detected by measuring Firefly luminescent signals. These values were normalized by Renilla luminescence values. The error bars show the standard error of the mean (SEM) from three independent experiments. Significance was confirmed by using unpaired two-tailed Student’s t-test. *p ≤ 0.05, **p ≤ 0.01, ***p ≤ 0.001.

In order to develop an AR functionality assay, experiments were conducted to select an AR lentiviral expression vector that can be used in co-transfection and co-transduction experiments in AR negative cell types. Three AR expression vectors were constructed with the AR open reading frames cloned into lentiviral expression vectors, *i*.*e*. pLenti6.3/AR-GC-E2325, pLenti6.3/ARV5-GC-E0060 and pLenti7.3/AR-E2325. These vectors were co-transfected with Cignal AR reporter in AR negative 293FT cells. The Cignal reporter consists of AR responsive Firefly luciferase and a constitutively expressing Renilla luciferase construct. The AR expression levels were detected in 293FT cells when induced with synthetic androgen 1 nM R1881 for 24 hours by using a dual-luciferase assay. This androgen-induced AR response effect was significantly higher with the pLenti6.3/AR-GC-E2325 vector construct (Fig 1). This vector construct was selected and further used in co-transfection and co-transduction experiments in AR negative cell lines.

### ARE reporter transfection and its response activity

The DBD of AR consists of two zinc fingers-like motifs that bind to the 15 base pairs palindromic repeat ARE consensus sequence [11, 31]. AREs are present in enhancer regions of androgen target genes. In order to develop an ARE based reporter assay, different combinations of ARE consensus promoter sequences were tested for the response and sensitivity to androgen-induced AR activity. Three ARE artificial promoter combinations, 241B, 248B and 249B were inserted in 3^rd^ generation lentiviral destination vector pEZ-Lv152. The ARE reporter vector CS-GS248B-gLUC-Lv152 contains the 248B promoter that was constructed by duplicating the ARE reporter sequence in the previously published (ARE)2-TATA Luc Reporter [32], thereby having 108 base pairs with 4 tandem repeats. The ARE reporter vector CS-GS249B-gLUC-Lv152 contains the 249B promoter that was generated by having four ARE tandem repeats of the ARE consensus sequence published previously [33]. The ARE reporter vector CS-GS241B-gLUC-Lv152 contains the 241B promoter that was constructed by selecting two out of three ARE consensus sequences found in the *KLK3* promoter region. AREI (AGAACAgcaAGTGCT) was inserted in four repeats and AREII (GGATCAgggAGTCTC) was inserted in two repeats, thereby having 109 base pairs with six ARE tandem repeats in total [34]. The sequences of the ARE consensus promoters are shown in Table 1. These vectors consist of ARE consensus artificial promoter fused to the mini-CMV promoter that drives Gaussia luciferase reporter. In addition, the vectors have an SV40 promoter-driven hygromycin selection marker (S1 Fig).

**Table 1:** ARE consensus promoter sequences.

ARE consensus promoter	Base pairs	ARE repeats	Sequence
241B	109	6	**GGATCAGGGAGTCTC**TCA**GGATCAGGGAGTCTC**AGT**AGAACAGCAAGTGCT**GTT**AGAACAGCAAGTGCT**GTA**AGAACAGCAAGTGCT**GCT**AGAACAGCAAGTGCT**GCGC
248B	108	4	CGGGAGCT**TGTACAGGATGTTCT**GCATGCTCTAGA**TGTACAGGATGTTCT**GGTACGGGAGCT**TGTACAGGATGTTCT**GCATGCTCTAGA**TGTACAGGATGTTCT**GGTA
249B	108	4	CGGGAGCT**AGAACANNNTGTTCT**GCATGCTCTAGA**AGAACANNNTGTTCT**GGTACGGGAGCT**AGAACANNNTGTTCT**GCATGCTCTAGA**AGAACANNNTGTTCT**GGTA

**Fig 2:**
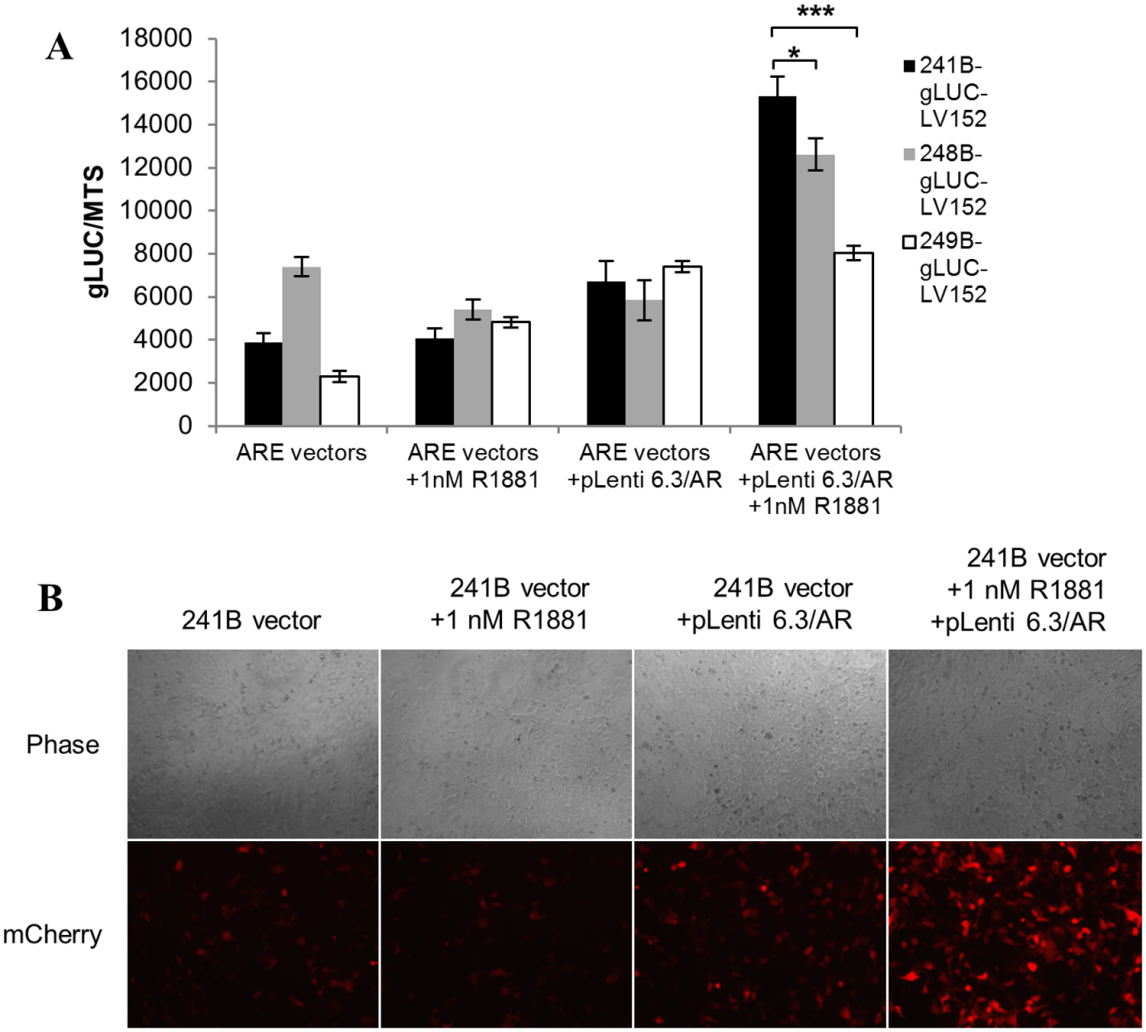
ARE promoter response and sensitivity to AR activity in 293FT cells. (A) Three different ARE promoter sequences, 241B, 248B and 249B inserted in a Gaussia luciferase reporter vector, were co-transfected with AR expression vector pLenti6.3/AR-GC-E2325 into 293FT cells. Cells were treated with 1 nM R1881 for 24 hours and Gaussia luciferase values were detected. These values were normalized by the MTS assay. (B) The 241B ARE promoter mCherry reporter vector was co-transfected with AR expression vector pLenti6.3/AR-GC-E2325 in 293FT cells. Fluorescence microscopy was performed after stimulating the cells with 1 nM R1881 for 24 hours. The error bars show the standard error of the mean (SEM) from three independent experiments. Significance was confirmed by using unpaired two-tailed Student’s t-test. *p ≤ 0.05, **p ≤ 0.01, ***p ≤ 0.001.

The ARE reporter vectors were co-transfected with the previously selected AR expression vector pLenti6.3/AR-GC-E2325 in 293FT cells. These cells were treated with 1 nM R1881 for 24 hours and the Gaussia luciferase response efficiencies of ARE reporter vectors were compared. MTS proliferation assays were performed to normalize the values. The luciferase reporter response in the co-transfected cells treated with R1881 showed a high AR specificity when compared to the cells that were only transduced with ARE reporters and treated with R1881. Additionally, the luciferase reporter response of the 241B consensus promoter showed a significantly higher sensitivity to androgen-induced AR activity in co-transfected cells treated with R1881 when compared among ARE reporter vectors (Fig 2A). The 241B promoter vector also showed a significantly higher difference in signal to noise ratio among the ARE reporter vectors when compared with non-treated cells that were co-transduced with ARE reporter and AR expression vector. Thus, the 241B promoter sequence reporter was selected among the constructed ARE reporters on the basis of its higher response activity and difference in desired signal to the level of background noise.

Afterwards, the 241B consensus promoter was cloned into pEZ-Lv152 with a mCherry fluorescent reporter, CS-GS241B-mCHER-Lv152 (S1 Fig). This vector consists of the 241B ARE consensus artificial promoter fused to the mini-CMV promoter that drives mCherry fluorescent signals. In addition, the vector has hygromycin selection marker attached to SV40 promoter. The 293FT cells were co-transfected with CS-GS241B-mCHER-Lv152 and pLenti6.3/AR-GC-E2325 AR expression vector. These cells were treated with 1 nM R1881 for 24 hours and the mCherry fluorescence response of the ARE reporter vector was analyzed by fluorescence microscopy. The mCherry fluorescent signal activity findings corresponded with luciferase reporter activity results (Fig 2B) and showed high fluorescent signal when compared with non-treated cells.

### ARE reporter responsive prostate cell lines

To generate ARE reporter responsive prostate cell lines, the AR positive and androgen responsive prostate cancer cell line, LNCaP [35], and the prostate basal transit amplifying epithelial cell line, EP156T-AR with exogenous AR [23], were transduced with CS-GS241B-mCHER-Lv152 vector. EP156T-AR cells were grown and maintained in androgen free MCDB medium, as ligand activation of exogenous AR in basal epithelial prostate cells induces growth arrest [36]. In previous attempts, cells were grown and maintained in regular MCDB153 medium that was not androgen-depleted for several passages. It was found that the AR expressing cells were selected against in androgen-containing MCDB153 medium (S3 Fig). In S3 Fig, the GFP signals show the transduction efficiency of AR in EP156T cells. However, AR expression was progressively attenuated during passage suggesting that AR expression is unstable in these cells and growth conditions. Recent studies confirm that the exogenous AR in the prostate basal epithelial cell line EP156T can induce androgen-dependent growth arrest [36, 37].

**Fig 3:**
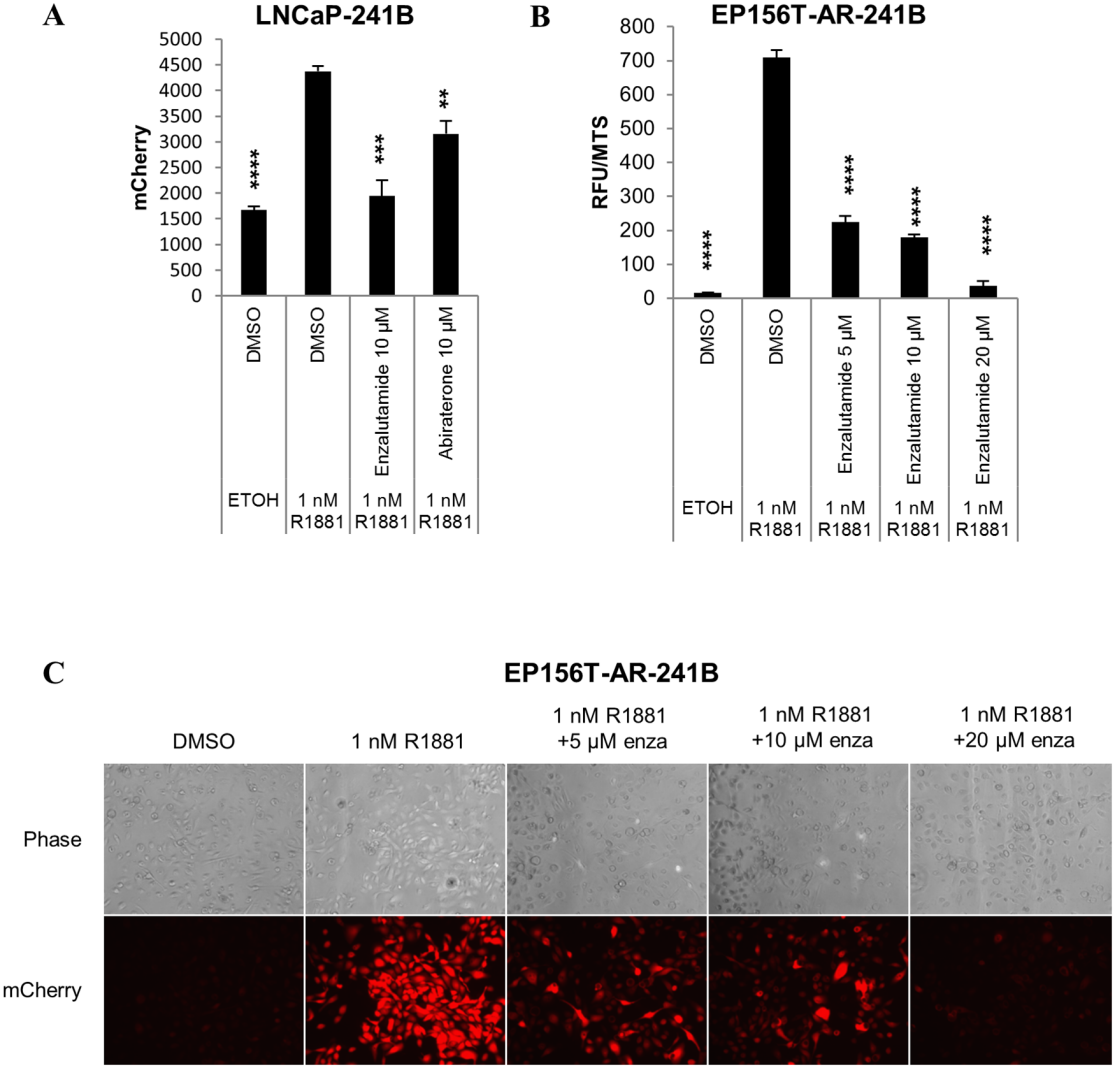
AR activity response in ARE reporter inserted prostate cells. (A) FACS analysis of mCherry fluorescent signals in LNCaP-241B cells grown in androgen free or supplemented with R1881 medium. The cells were treated with 10 μM enzalutamide and 10 μM abiraterone for 24 hours. (B) Relative fluorescence units (RFU) measurements of mCherry signals in EP156T-AR-241B cells grown in androgen free growth medium in 96-well plates. Cells were treated with ± 1 nM R1881 and increasing concentrations of enzalutamide, *i*.*e*. 5 μM, 10 μM and 20 μM for 24 hours. The RFU values were normalized by the MTS assay. (C) Fluorescence microscopy of EP156T-AR-241B cells treated with ± 1 nM R1881 and different concentrations of enzalutamide (enza), *i*.*e*. 5 μM, 10 μM and 20 μM for 24 hours. The error bars show the standard error of the mean (SEM) from three independent experiments. Significance was confirmed by using unpaired two-tailed Student’s t-test. *p ≤ 0.05, **p ≤ 0.01, ***p ≤ 0.001, ****p ≤ 0.0001.

The generated LNCaP-241B and EP156T-AR-241B cell lines showed androgen-dependent reporter responsiveness when treated with 1 nM R1881 according to fluorescence microscopy, flow cytometry and multi-well fluorescent recording (Fig 3). Fluorescence microscopy results may be subjective; therefore, flow cytometry and multi-well fluorescent recording were used to quantify the signals. To assess if the androgen led reporter activity can be neutralized, the androgen stimulated LNCaP-241B reporter cells were treated with 10 μM of the AR antagonist, enzalutamide and 10 μM of the anti-androgen abiraterone. Enzalutamide is a second generation AR antagonist approved by the USA Food and Drug Administration (FDA) in 2012 for the therapy of CRPC [38]. Enzalutamide binds to the LBD of AR with high affinity and restricts AR nuclear import, hinders co-activator recruitment and reduces the AR binding efficiency to ARE in the nucleus [39, 40]. Abiraterone is an inhibitor of cytochrome P17 (17 α-hydroxylase/C17,20-lyase) enzyme referred to as CYP17, and inhibits androgen biosynthesis in the adrenal glands, testes and within the prostate cancer. It was approved in 2011 by the FDA for the treatment of late-stage CRPC [41, 42]. As expected, the ARE-driven reporter showed a much stronger reduction in mCherry fluorescent signals in LNCaP-241B cells when treated with enzalutamide as compared to abiraterone (Fig 3A), as is also evident by fluorescence microscopy (S4 Fig). Although abiraterone primarily is an androgen synthesis inhibitor, it caused a decrease in ARE reporter response, although not as pronounced as enzalutamide. Recent studies suggest that abiraterone also impairs AR nuclear import in the presence of R1881 [43]. In addition, androgen stimulated EP156T-AR-241B reporter cells showed a gradual decrease in mCherry fluorescence activity when treated with increasing doses of enzalutamide both according to the fluorescence plate reader (Fig 3B) and microscopy (Fig 3C). The Relative fluorescence units (RFU) from the fluorescence plate reader were normalized by MTS proliferation assays.

Next, we evaluated the robustness of our ARE-reporter based AR activity assay using a statistical parameter Z´-factor assay [25]. Z´-factor is a quantitative measure of the separation between the baseline and induced reporter activation. If the assay’s Z´-factor ≥ 0.5, it is consistent with a good quality assay. The Z´-factor values of our generated reporter cell lines EP156T-AR-241B, 957E/hTERT-AR-241B and LNCaP-241B were 0.89, 0.93 and 0.88, respectively, following induction of reporter expression by 1 nM R1881. The calculated Z´-factor values of each cell line indicate excellent quality of the assays.

### Application of GFP as an internal normalization in reporter cells

**Fig 4:**
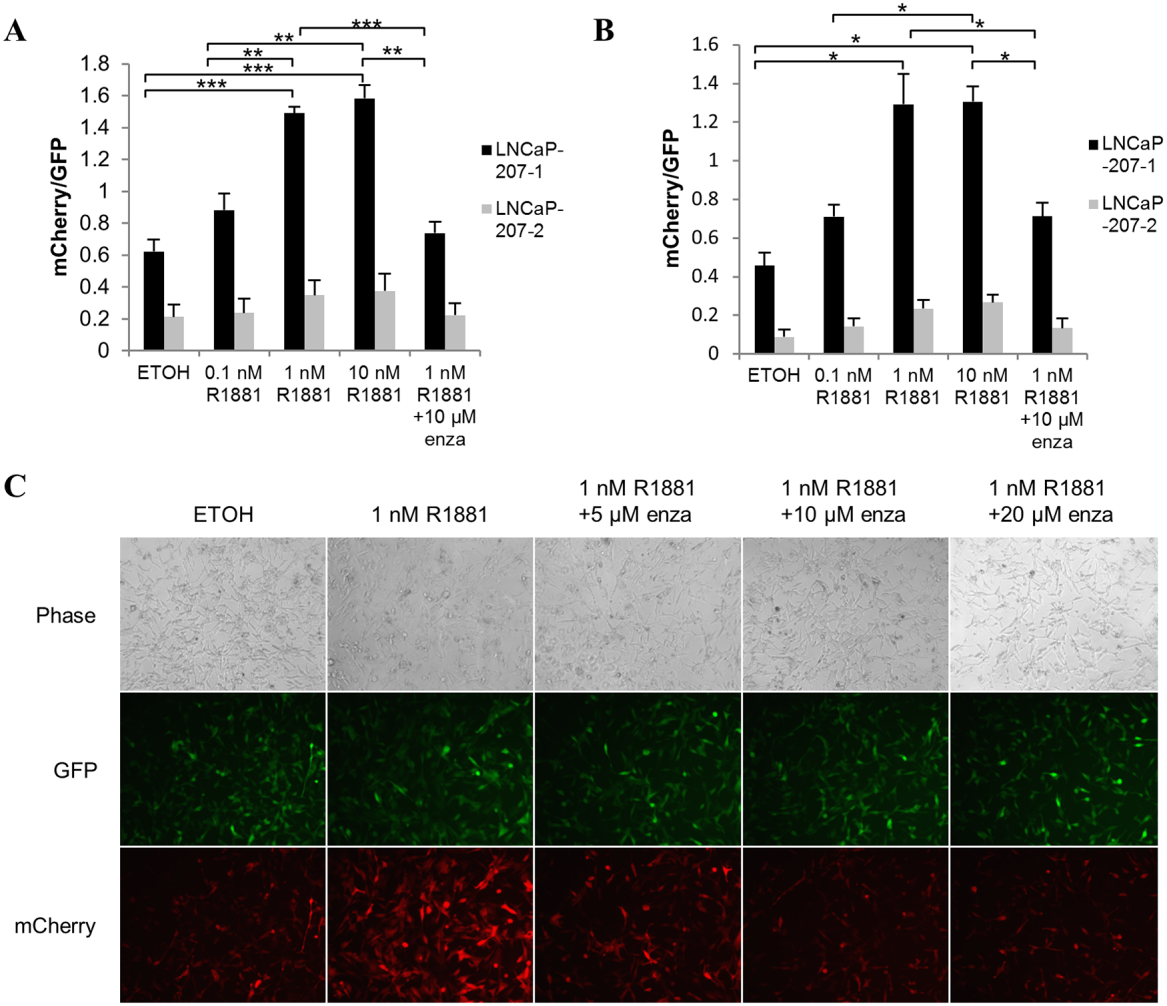
Internal normalization of ARE reporter signals in prostate cells. (A) FACS analysis of mCherry fluorescent signals in LNCaP-207-01 and LNCaP-207-02 cells grown in androgen free medium. Cells were treated with different concentrations of androgen R1881, *i*.*e*. 0.1 nM, 1 nM and 10 nM, and 10 μM enzalutamide (enza) with 1 nM R1881 for 24 hours. The values were normalized by constitutively expressed GFP fluorescent signals. (B) Multi-well mCherry RFU measurements of LNCaP-207-01 and LNCaP-207-02 cells grown in androgen free medium. The cells were treated with different concentrations of androgen R1881, *i*.*e*. 0.1 nM, 1 nM and 10 nM, and 10 μM enzalutamide with 1 nM R1881 for 24 hours. The mCherry RFU values were normalized by GFP RFU. (C) Fluorescence microscopy of LNCaP-207-01 cells treated with ± 1 nM R1881 and different concentrations of enzalutamide, *i*.*e*. 5 μM, 10 μM and 20 μM for 24 hours. The error bars show the standard error of the mean (SEM) from three independent experiments. Significance was confirmed by using unpaired two-tailed Student’s t-test. *p ≤ 0.05, **p ≤ 0.01, ***p ≤ 0.001.

In order to develop a fast track ARE reporter system that will facilitate the investigation of AR functional activity, an internal normalization system of ARE driven mCherry fluorescent signals was developed. For this purpose, the constitutively expressing eGFP reporter vector, CS-GS241B-mCHER-Lv207-01 was transduced into LNCaP cells to generate LNCaP-207-01 reporter cells. In this vector the 241B ARE promoter sequence was cloned into a 3rd generation lentiviral mCherry reporter vector pEZ-Lv152 with the eGFP and hygromycin resistance genes driven by the SV40 promoter and separated by an internal ribosome entry site (IRES) as shown in S1 Fig. LNCaP-207-01 reporter cells showed a stable eGFP signals and increased in ARE driven mCherry response when treated with synthetic androgen R1881 that could be neutralized dose dependently by enzalutamide (Fig 4C). We wanted to develop a reporter system that can extensively investigate the AR functional activity in multi-well fluorescent recording. The ARE reporter in LNCaP-207-01 cells showed a similar response pattern in FACS analysis (Fig 4A) compared to multi-well fluorescence reader (Fig 4B) when treated with different concentrations of R1881 and in combination with enzalutamide. Although there are some limitations in sensitivity of multi-well readings when it comes to fluorescent signal measurements as compared to FACS, the fluorescence reporter response can be measured in both systems.

In an effort to further increase the mCherry fluorescence signal to noise ratio. LNCaP cells were transduced with the CS-GS241B-mCHER-Lv207-02 ARE reporter vector (LNCaP-207-02) that contained DNA core insulator enhancers (CIEs) in addition to constitutively expressing eGFP and hygromycin resistance genes as shown in S1 Fig. These DNA insulator elements are specialized DNA sequences that were initially found in *Drosophila*, and prevent external enhancers from improper activation of reporter gene promoters [44, 45]. These sequences prevent cross-regulation of adjacent genes and protect against position effects of recombinant genes [46]. The addition of CIEs in LNCaP-207-02 cells reduced the fluorescence bleed-through in untreated cells, however, it also reduced the androgen-induced responsiveness of ARE driven mCherry fluorescent signals when compared to LNCaP-207-01 reporter cells treated with different doses of the synthetic androgen R1881 as shown in Fig 4A and 4B. It is likely that CIEs interfered with enhancer-promoter communication and reduced the expression of the reporter gene. Some studies show that the insulator sequences compete for enhancers and disrupt transcriptional activation [47].

### Differential ARE reporter response to different steroid hormones

**Fig 5:**
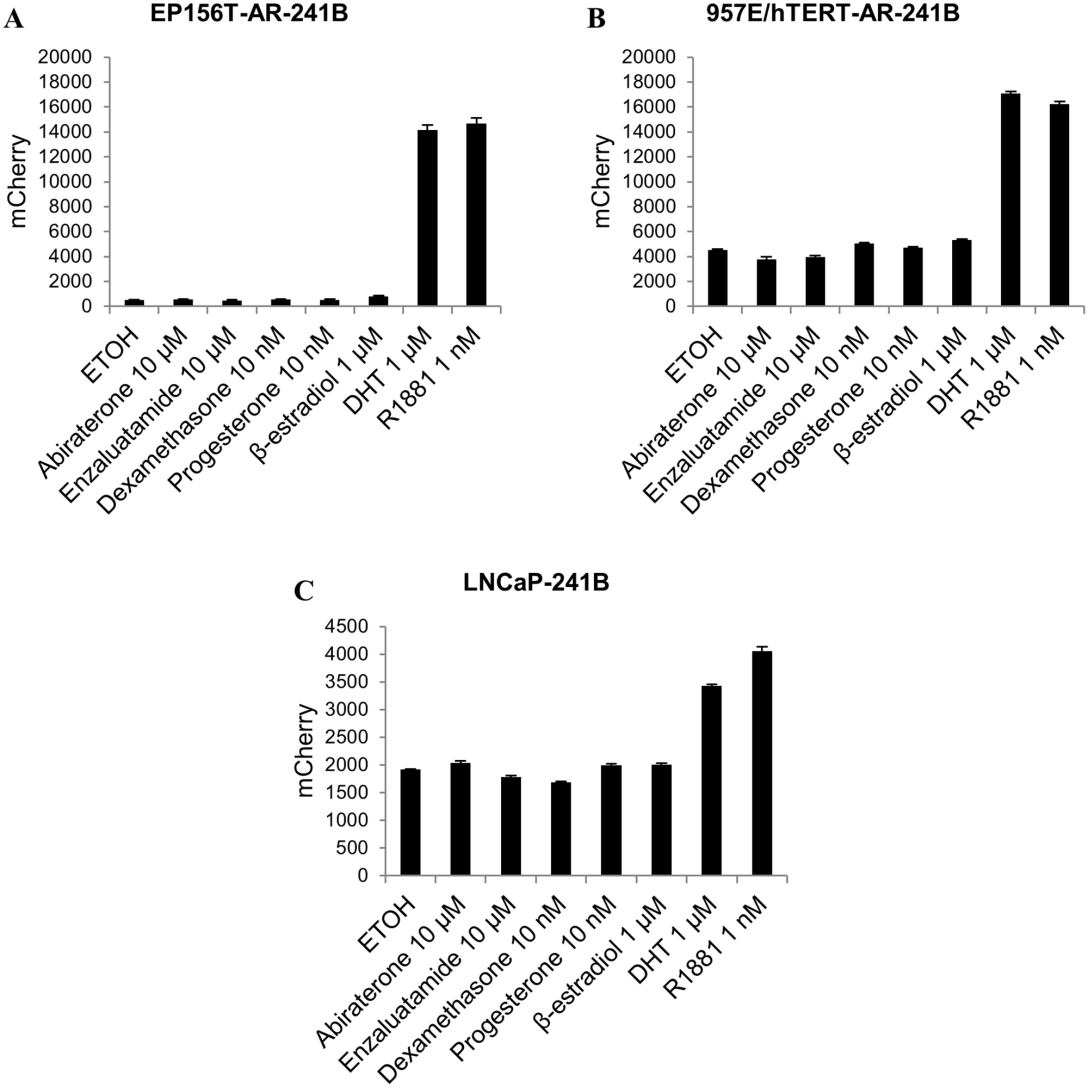
ARE reporter response to different steroids. FACS analysis of mCherry signals in (A) EP156T-AR-241B, (B) 957E/hTERT-AR-241B and (C) LNCaP-241B cells. Cells were grown in androgen free medium and treated with different AR agonists (1 nM R1881 and 1 μM DHT), steroids (10 nM dexamethasone, 10 nM progesterone and 1 μM β-estradiol), AR antagonist (10 μM enzalutamide) and anti-androgen (10 μM abiraterone). The error bars show the standard deviation (SD) from three independent experiments.

Ligand binding to AR is the primary and critical step of ligand-dependent transcription of AR target genes. The three-dimensional structure of AR LBD shares some commonalities with other ligand-bound steroid receptors [48]. Steroid hormones retain a certain degree of structural similarities that may exhibit androgenic responses. For example, β-estradiol, progesterone and dexamethasone show some level of affinity to the AR [49]. The ARE reporter activity was tested in generated cell lines when treated with different androgen agonists and other steroids. In Fig 5, the reporter cell lines EP156T-AR-241B (Fig 5A), 957E/hTERT-AR-241B (Fig 5B) and LNCaP-241B (Fig 5C) showed significantly higher reporter activity when treated with AR agonists, R1881 and DHT compared to when treated with other steroids, *i*.*e*. progesterone, β-estradiol and dexamethasone. This demonstrates a high androgen-specificity of the ARE reporter. Studies suggest that the most frequent mutation of the *AR* gene in androgen-independent human prostate cancer LNCaP cells found is the substitution of amino acid T877A in the AR LBD. This mutant AR increases the binding affinity of other steroids such as progesterone, β-estradiol and glucocorticoid. It results in inappropriate activation of AR transcriptional activity [50, 51]. Interestingly, the ARE reporter response in LNCaP cells (Fig 5C) shows very little or no reporter response to steroid-mediated AR activation, suggesting no significant transcriptional activation of classical luminal differentiation AR target genes as compared to conventional androgen-led AR activation. In comparison to different studies that suggest β-estradiol-mediated AR activation can lead to *PSA* gene transactivation [52, 53], our result suggests that β-estradiol is unable to activate the ARE reporter.

### Different AR status in different prostate cells affects the ARE-mCherry reporter

With the introduction of the ARE reporter system in different prostate cell lines, we have observed some basal level of mCherry fluorescence activity in these cell lines. This basal fluorescence level varies among the generated prostate reporter cell lines when they were grown in androgen-free conditions. The degree of basal fluorescence level in the cell lines can range from a background and/or bleed-through fluorescent signals to a certain level of AR activity in androgen depleted conditions.

**Fig 6:**
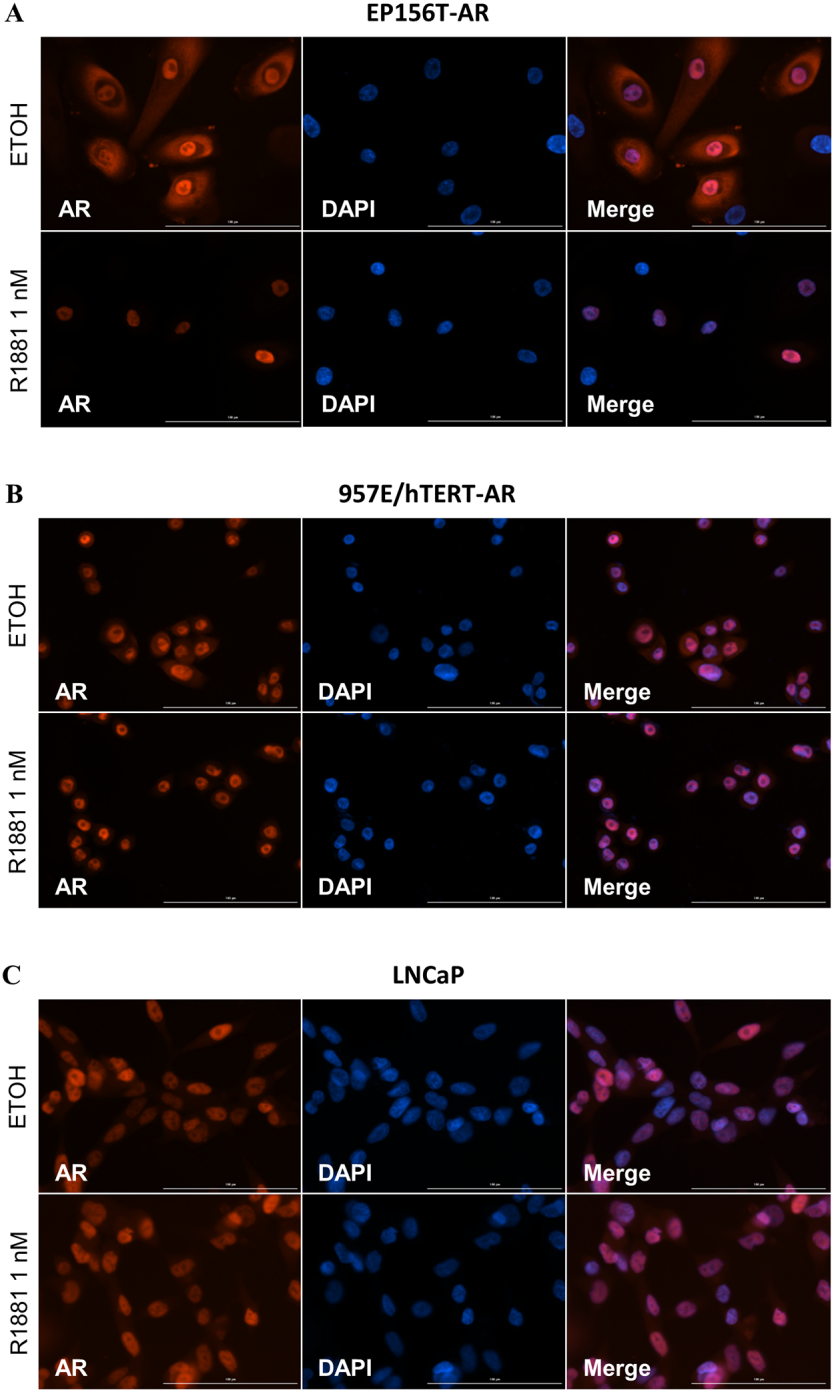
AR protein localization in prostate cells. Texas red (Tx-Red) indirect immunofluorescent detection of AR in (A) EP156T-AR, (B) 957E/hTERT-AR and (C) LNCaP cells. The cells were treated with ± 1 nM R1881 for 24 hours.

Fig 5 indicates a lower basal fluorescence level in EP156T-AR-241B than in 957E/hTERT-AR-241B and LNCaP-241B cells when the cells were grown in androgen-free medium. Interestingly, these findings corresponded with AR nuclear localization in the respective cell lines. As shown in Fig 6, the AR has less nuclear localization in EP156T-AR cells in androgen-free medium (Fig 6A) than in 957E/hTERT-AR (Fig 6B) and LNCaP cells (Fig 6C). LNCaP cells are also reported to have increased AR expression and a basal AR transcriptional activity in androgen-depleted conditions [54]. Studies have also shown ligand-independent activation of AR by growth factors and protein kinase A activators [55, 56]. Additionally, there might still be very low levels of steroids present in CS FCS. The higher basal reporter activity in LNCaP-241B cells may support the concept of AR hypersensitivity and hyperactivity development in androgen-free conditions.

Some degree of background fluorescence and bleed-through may exist in the generated prostate reporter cell lines. These signals can also be observed when AR negative DU145 prostate cancer cells and EP156T cells were transduced with 241B ARE-reporter system (data not shown). Thus, our findings suggest that in spite some background noise signals low AR activity signals might persist in androgen-depleted conditions.

### Exogenous AR does not lead to ARE reporter response in mesenchymal type cells

**Fig 7:**
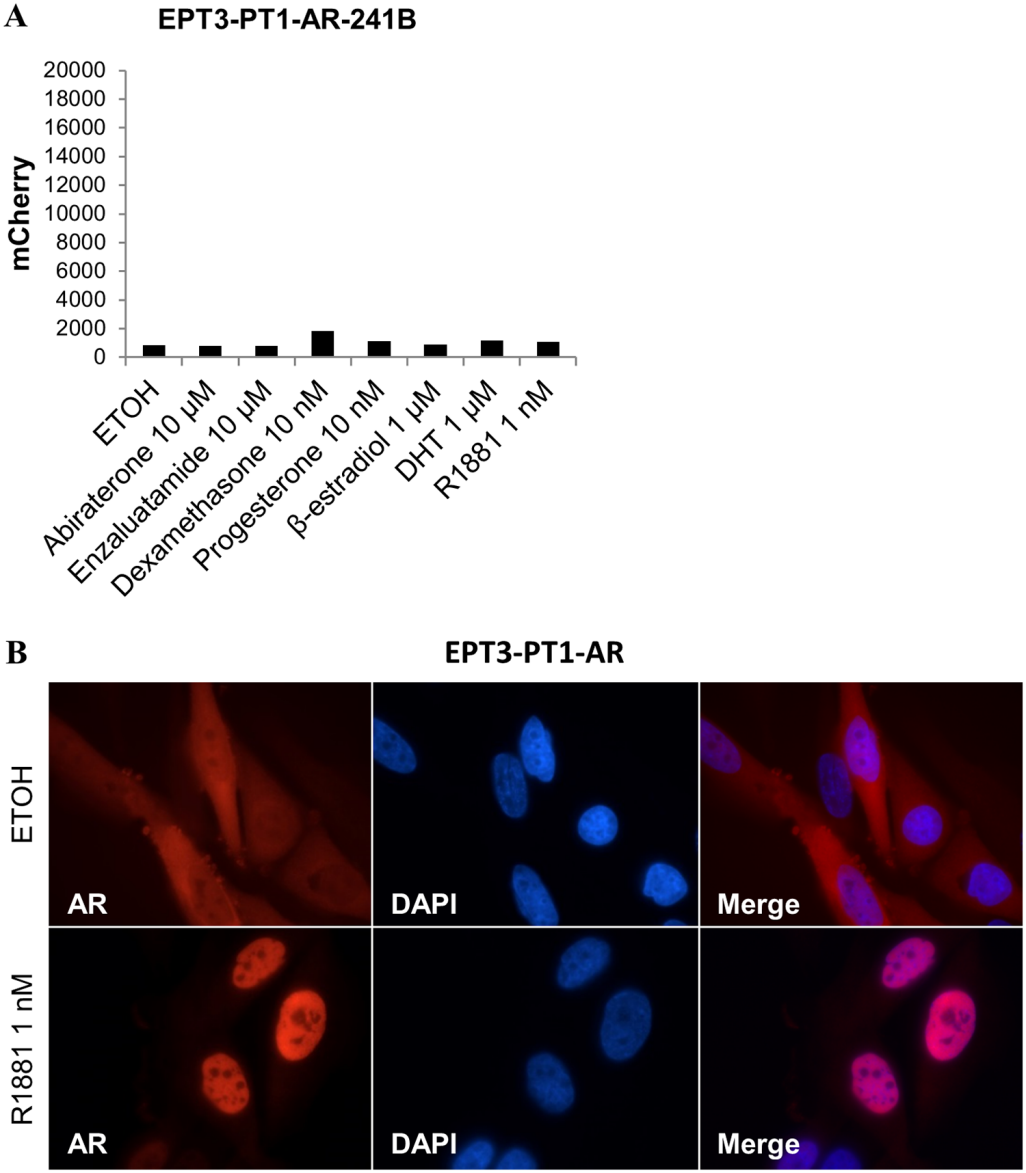
ARE reporter response in mesenchymal type cells. (A) FACS analysis of mCherry signals in EPT3-PT1-AR-241B cells. Cells were grown in androgen free medium and treated with different AR agonists (1 nM R1881 and 1 μM DHT), steroids (10 nM dexamethasone, 10 nM progesterone and 1 μM β-estradiol), AR antagonist (10 μM enzalutamide) and anti-androgen (10 μM abiraterone). (B) Texas red (Tx-Red) indirect immunofluorescent detection of AR in EPT3-PT1-AR-241B cells. The cells were treated with ± 1 nM R1881 for 24 hours.

We also investigated the ARE reporter response in the mesenchymal type EPT3-PT1 cells. We have previously published the stepwise generation of malignant prostate, mesenchymal EPT3-PT1 cells from benign epithelial EP156T cells by physiological adaptation and selection [23, 57–59]. In spite of detectable AR in EPT3-PT1 cells compared to its progenitor AR negative EP156T cells, EPT3-PT1 cells showed no androgen-dependent induction of AR and its classical luminal differentiation target genes. EPT3-PT1 prostate cells were transduced with AR expression vector to generate EPT3-PT1-AR as published previously [37]. The generated EPT3-PT1-AR cells express AR at a level comparable to LNCaP cells. When these cells were transduced with the 241B ARE reporter, EPT3-PT1-AR-241B cells showed no reporter response (Fig 7A). This suggests that the ARE-reporter in mesenchymal type EPT3-PT1-AR-241B cells is androgen non-responsive, in contrast to in epithelial type EP156T-AR-241B cells. Interestingly, EPT3-PT1-AR cells demonstrated androgen-dependent AR nuclear import as shown in Fig 7B. A complete AR shift to the nucleus was observed when 1 nM R1881 was added. These findings also corroborated our previous findings, where the exogenous expression of AR in mesenchymal type EPT3-PT1-AR cell was unable to induce classical AR target genes [37]. Finally, this demonstrates a high specificity and sensitivity of the ARE reporter for androgen-mediated AR functional activity.

## Conclusions

The pLenti6.3/AR-GC-E2325 androgen receptor expression vector was selected in initial screenings on the basis of high-level exogenous AR expression. This AR expression vector was further used in co-transfection and co-transduction experiments in AR negative cells. The ARE promoter sequence 241B was found to have high sensitivity reporter response to an androgen-dependent AR activity among several generated ARE promoter sequences. When the 241B ARE promoter was linked to the mCherry fluorescence reporter, the signals were measured and quantified using flow cytometry and multi-well fluorescence reader on the basis of AR transcriptional activity. The prostate cell lines with the 241B ARE promoter mCherry reporter easily detected AR transcriptional activity in a multi-well fluorescent recorder. The AR antagonists or drugs affecting AR signaling activity can be analyzed by the relative quantification of the mCherry reporter response. The addition of GFP as an internal control in the reporter abolished the need to use other means for normalization such as MTS which requires more experiments and can be biased by drugs or stimulations that affect cell metabolism. The developed system demonstrated a strong specificity to androgen-mediated AR transcriptional activity compared to other steroid hormones such as β-estradiol, progesterone and dexamethasone. Expression of exogenous AR in epithelial type cells induced an androgen-dependent ARE reporter response, however, exogenous AR expression were unable to induce ARE reporter response in mesenchymal type cells. These findings also conclude that exogenous AR expression was able to induce a classical androgen-dependent AR transcriptome in, such as KLK3, KLK2 FKBP5, in epithelial type cells but not in mesenchymal type cells. This developed reporter response system can be useful in drug screening and will also help to investigate the role of the AR in terminal differentiation of prostate cells both in two and three-dimensional cell cultures.

## Supporting information

S1 Fig ARE reporter vectors schematic maps. (A) The ARE promoters 241B, 248B or 249B fused to the mini-cytomegalovirus (mCMV) promoter drive Gaussia luciferase expression and Simian virus 40 promoter (SV40) facilitates constitutive expression of the *hygromycin* resistance gene. (B) The ARE sequences 241B, 248B or 249B fused to the mCMV promoter drive mCherry expression, and the SV40 promoter facilitates constitutive expression of the *hygromycin* resistance gene. (C) The ARE sequence 241B fused to mCMV promoter drives mCherry expression, and SV40 promoter facilitates constitutive expression of *eGFP* and *hygromycin* resistance genes that are separated by an internal ribosome entry site (IRES). (D) The ARE sequence 241B fused to mCMV promoter drives mCherry expression. The core insulator enhancers (CIE) are added on each side. SV40 promoter facilitates constitutive expression of *eGFP* and *hygromycin* resistance gene that are separated by an IRES.
(TIF)

S2 Fig Schematics of AR expression vectors. (A) The pLenti6.3/ARV5-GC-E0060 vector contains the human cytomegalovirus (CMV) promoter that ensures constitutive high level expression of the downstream AR-V5 tag gene GC-E0060. The vector contains the *blasticidin* resistance gene driven by the SV40 promoter. (B) The pLenti7.3/ARV5-GC-E2325 vector contains the CMV promoter that ensures constitutive high level expression of the downstream *AR* gene GC-E2325. The vector contains the eGFP marker driven by the SV40 promoter.
(TIF)

S3 Fig Exogenous AR expression and nuclear import. Immunofluorescence (A) EP156T cells were transduced with the pLenti7.3/AR-E2325 vector that allows constitutive exogenous expression of AR with eGFP as a marker protein to generate EP156T-AR cells. After transduction and eGFP selection, the cells were maintained in regular MCDB153 medium for several passages. The cells were plated and treated with ± 1 nM R1881 for 48 hours. Texas red (Tx-Red) fluorescent signals indicate AR. (B) EP156T cells were transduced with pLenti6.3/AR-E2325 vector that allows constitutive exogenous expression of AR to generate EP156T-AR cells. After transduction and blasticidin selection, the cells were maintained in regular MCDB153 medium for several passages. The cells were treated with ± 1 nM R1881 for 48 hours. FITC fluorescent signals indicate AR.
(TIF)

S4 Fig AR activity reporter response in LNCaP-241B cells. Fluorescence microscopy of mCherry fluorescent signals in LNCaP-241B cells grown in androgen free medium or supplemented with R1881. Treatment with 10 μM enzalutamide or 10 μM abiraterone was for 24 hours.
(TIF)
