# Supplementary figures and images for: An androgen response element driven reporter assay for the detection of androgen receptor activity in prostate cells
Source: PLoS One. 2017 Jun 1;12(6):e0177861. doi: 10.1371/journal.pone.0177861 (PMC5453475; doi:10.1371/journal.pone.0177861)

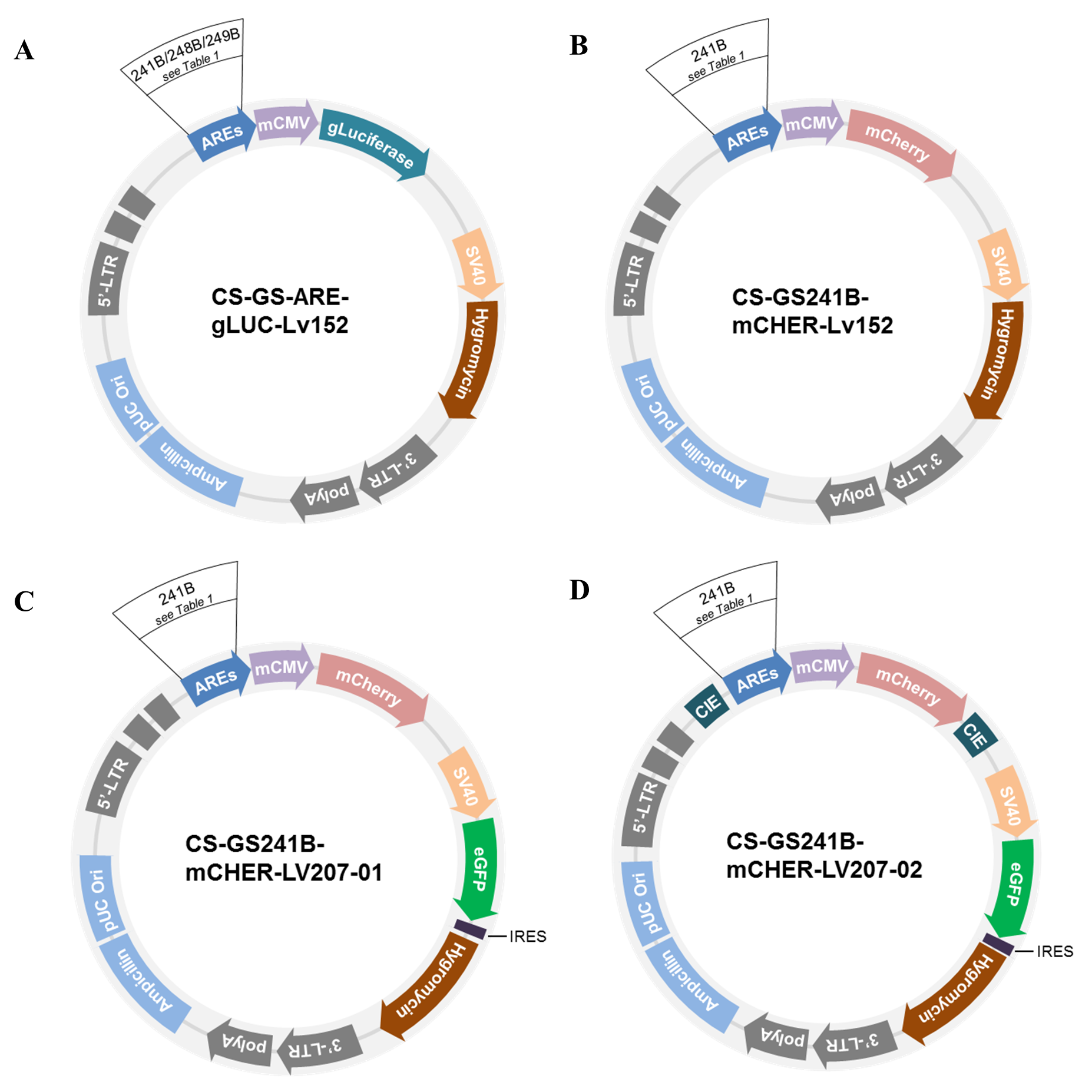

Supplement: S1 Fig — (A) The ARE promoters 241B, 248B or 249B fused to the mini-cytomegalovirus (mCMV) promoter drive Gaussia luciferase expression and Simian virus 40 promoter (SV40) facilitates constitutive expression of the hygromycin resistance gene. (B) The ARE sequences 241B, 248B or 249B fused to the mCMV promoter drive mCherry expression, and the SV40 promoter facilitates constitutive expression of the hygromycin resistance gene. (C) The ARE sequence 241B fused to mCMV promoter drives mCherry expression, and SV40 promoter facilitates constitutive expression of eGFP and hygromycin resistance genes that are separated by an internal ribosome entry site (IRES). (D) The ARE sequence 241B fused to mCMV promoter drives mCherry expression. The core insulator enhancers (CIE) are added on each side. SV40 promoter facilitates constitutive expression of eGFP and hygromycin resistance gene that are separated by an IRES. (TIF) [file pone.0177861.s001.tif]

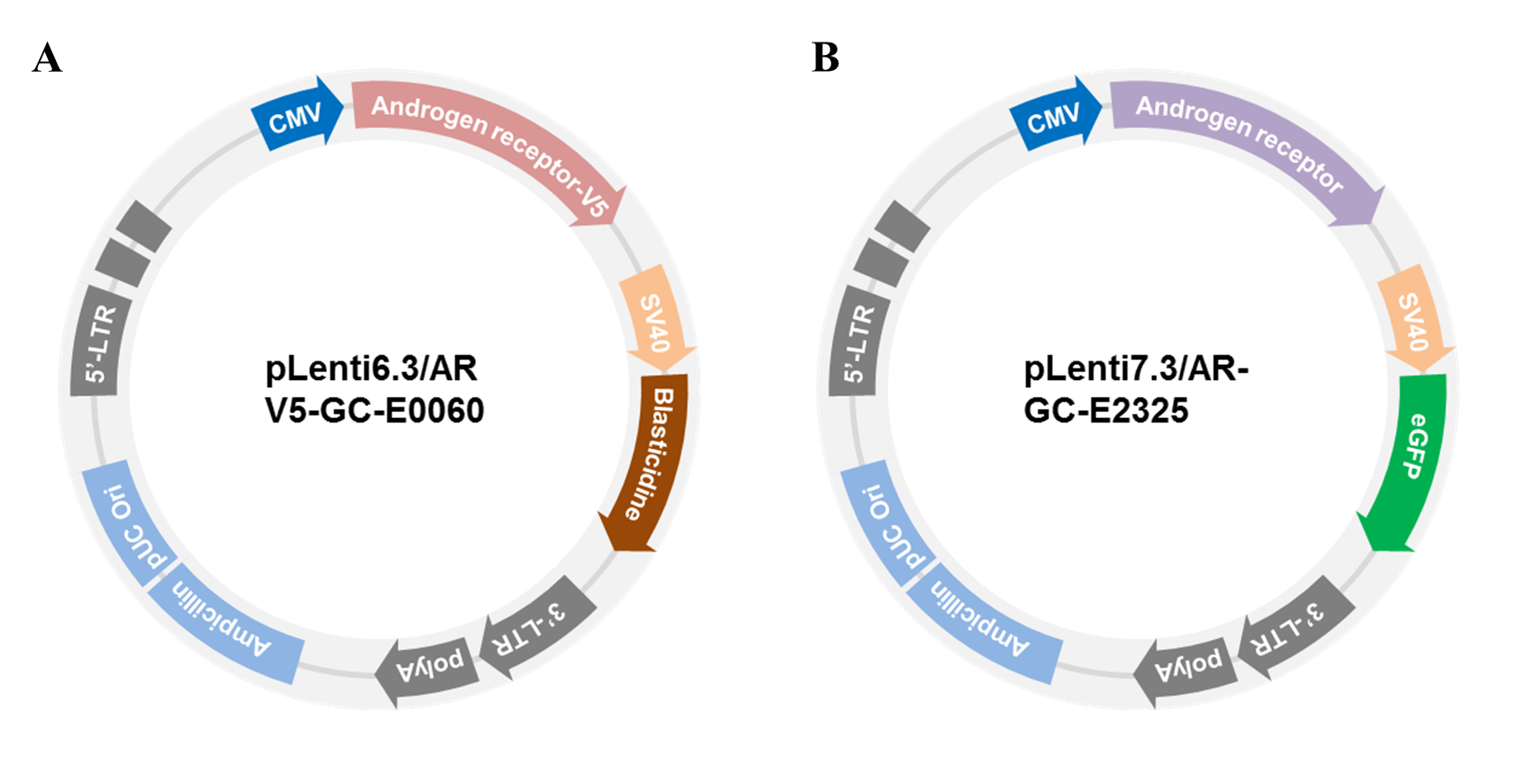

Supplement: S2 Fig — (A) The pLenti6.3/ARV5-GC-E0060 vector contains the human cytomegalovirus (CMV) promoter that ensures constitutive high level expression of the downstream AR-V5 tag gene GC-E0060. The vector contains the blasticidin resistance gene driven by the SV40 promoter. (B) The pLenti7.3/ARV5-GC-E2325 vector contains the CMV promoter that ensures constitutive high level expression of the downstream AR gene GC-E2325. The vector contains the eGFP marker driven by the SV40 promoter. (TIF) [file pone.0177861.s002.tif]

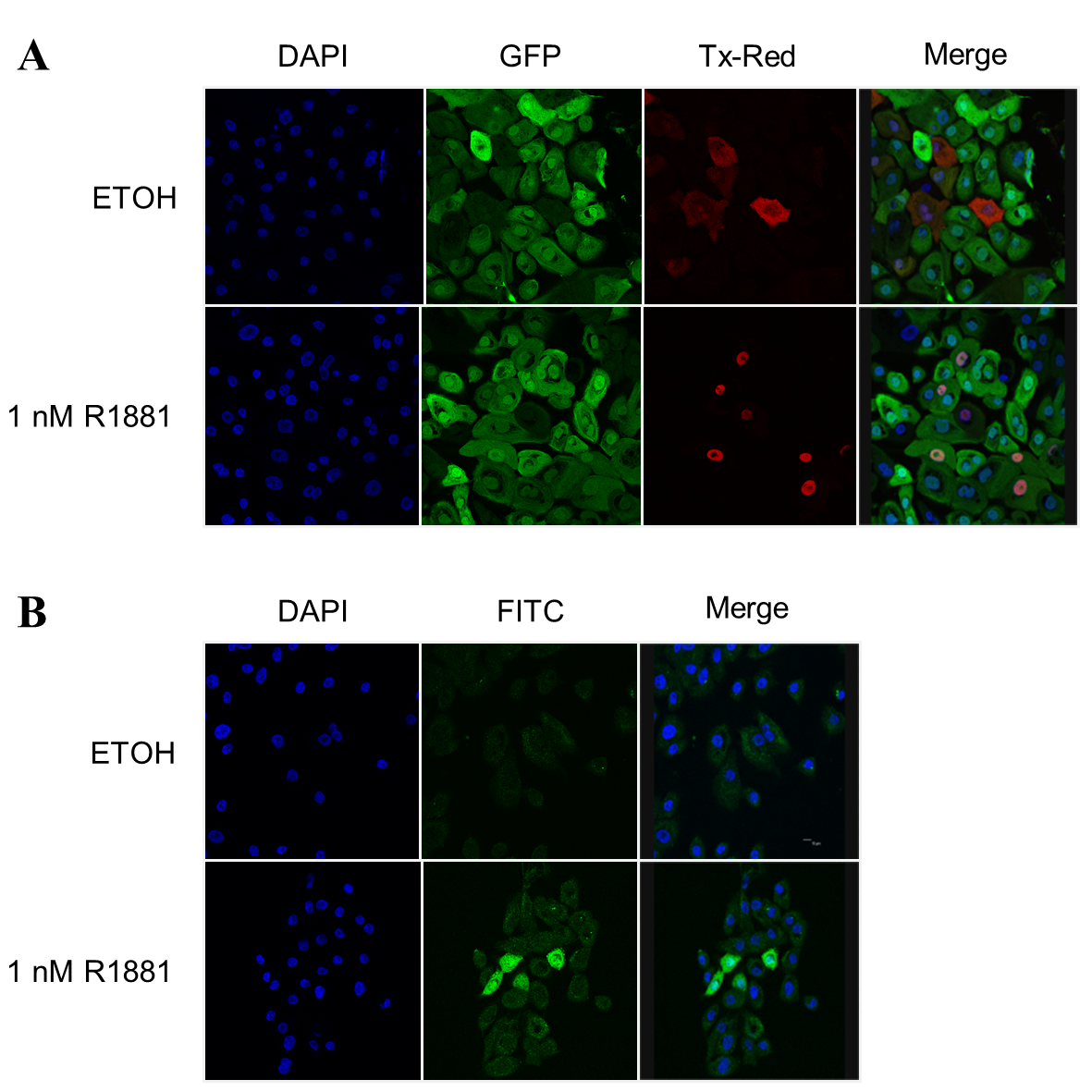

Supplement: S3 Fig — Immunofluorescence (A) EP156T cells were transduced with the pLenti7.3/AR-E2325 vector that allows constitutive exogenous expression of AR with eGFP as a marker protein to generate EP156T-AR cells. After transduction and eGFP selection, the cells were maintained in regular MCDB153 medium for several passages. The cells were plated and treated with ± 1 nM R1881 for 48 hours. Texas red (Tx-Red) fluorescent signals indicate AR. (B) EP156T cells were transduced with pLenti6.3/AR-E2325 vector that allows constitutive exogenous expression of AR to generate EP156T-AR cells. After transduction and blasticidin selection, the cells were maintained in regular MCDB153 medium for several passages. The cells were treated with ± 1 nM R1881 for 48 hours. FITC fluorescent signals indicate AR. (TIF) [file pone.0177861.s003.tif]

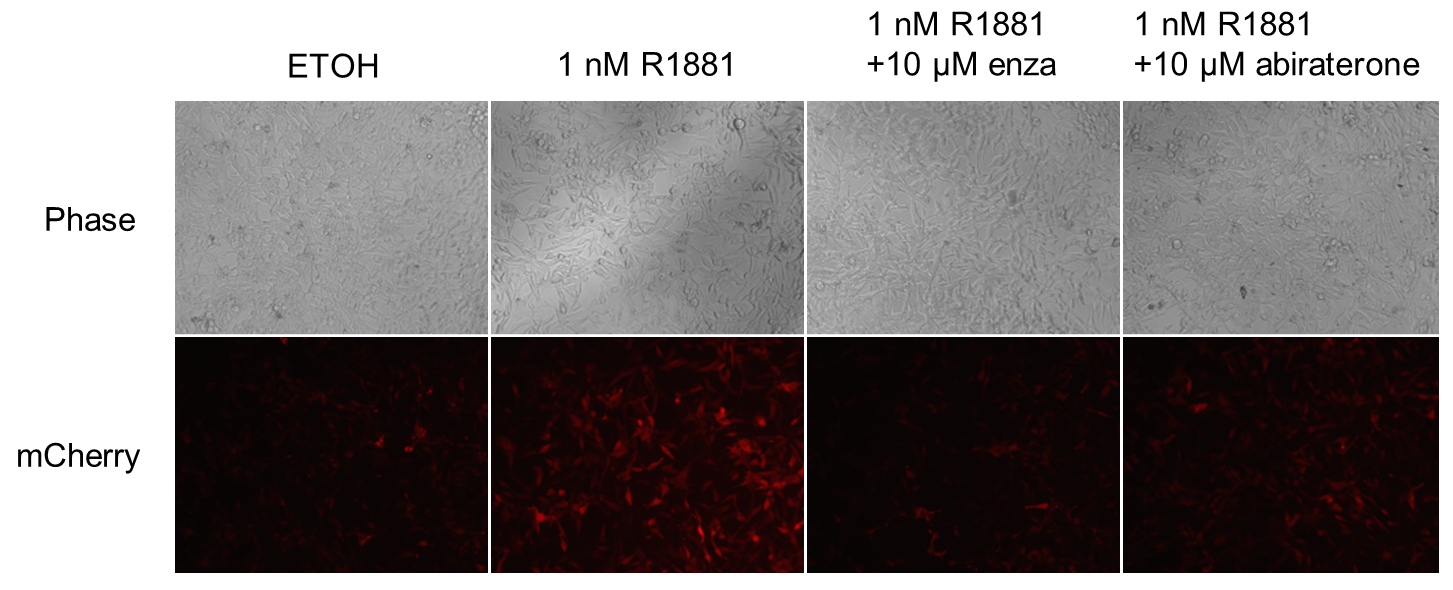

Supplement: S4 Fig — Fluorescence microscopy of mCherry fluorescent signals in LNCaP-241B cells grown in androgen free medium or supplemented with R1881. Treatment with 10 μM enzalutamide or 10 μM abiraterone was for 24 hours. (TIF) [file pone.0177861.s004.tif]
